# Radiation biology of mosquitoes

**DOI:** 10.1186/1475-2875-8-S2-S6

**Published:** 2009-11-16

**Authors:** Michelle EH Helinski, Andrew G Parker, Bart GJ Knols

**Affiliations:** 1 Entomology Unit, FAO/IAEA Agriculture and Biotechnology Laboratory, Joint FAO/IAEA Programme, International Atomic Energy Agency, Vienna, Austria; 2 Medical Entomology, Cornell University, 3136 Comstock Hall, Ithaca, NY 14853-2601, USA; 3 Div. Infectious Diseases, Tropical Medicine & AIDS, Academic Medical Center, F4-217, Meibergdreef 9, 1105 AZ Amsterdam, The Netherlands and K&S Consulting, Kalkestraat 20, 6669 CP Dodewaard, The Netherlands

## Abstract

There is currently renewed interest in assessing the feasibility of the sterile insect technique (SIT) to control African malaria vectors in designated areas. The SIT relies on the sterilization of males before mass release, with sterilization currently being achieved through the use of ionizing radiation. This paper reviews previous work on radiation sterilization of *Anopheles *mosquitoes. In general, the pupal stage was irradiated due to ease of handling compared to the adult stage. The dose-response curve between the induced sterility and log (dose) was shown to be sigmoid, and there was a marked species difference in radiation sensitivity. Mating competitiveness studies have generally been performed under laboratory conditions. The competitiveness of males irradiated at high doses was relatively poor, but with increasing ratios of sterile males, egg hatch could be lowered effectively. Males irradiated as pupae had a lower competitiveness compared to males irradiated as adults, but the use of partially-sterilizing doses has not been studied extensively. Methods to reduce somatic damage during the irradiation process as well as the use of other agents or techniques to induce sterility are discussed. It is concluded that the optimal radiation dose chosen for insects that are to be released during an SIT programme should ensure a balance between induced sterility of males and their field competitiveness, with competitiveness being determined under (semi-) field conditions. Self-contained ^60^Co research irradiators remain the most practical irradiators but these are likely to be replaced in the future by a new generation of high output X ray irradiators.

## Background

The sterile insect technique (SIT) for mosquitoes includes the mass production, sex separation, sterilization and release of sterile males. Contemporary methods available to induce sterility in the released insects are ionizing radiation or chemosterilization. Chemosterilants were used experimentally and in field trials in the 1960-70s against mosquitoes [1,2] but they were mutagenic, and thus presented a potential hazard to humans during the treatment process (but see [3]). Their use was discontinued after concerns were raised about the effect of residues in the environment and on non-target organisms, particularly when large numbers of treated insects were released [4]. These concerns were based mainly on the findings of one, so far un-replicated, study that found that spiders fed on a diet of only chemosterilized mosquitoes subsequently became sterile [5]. Although the amount of residue released in the environment was very low, due to the careful rinsing of pupae [6], ionizing radiation has become the principal technique for sterilization, even though it has been reported to reduce competitiveness of the males more than chemosterilization [2,7]. However, successful SIT programmes for the elimination of the New World screwworm *Cochliomyia hominivorax *from the USA, Central America [8] and Libya [9] and the tsetse fly *Glossina austensi *from Zanzibar [10] relied on radiation-sterilized insects, as well as the ongoing SIT programmes against the Mediterranean fruit fly *Ceratitis capitata *from Central and Latin America [11].

A variety of novel sterilization methods based on transgenesis are currently under development [12,13] and are discussed in detail in [14]. However, for mosquitoes, many of these technologies are still in the experimental phase and little regulatory framework exists for the introduction of transgenic mosquitoes into the wild [15]. The aim of this paper is to give an overview of irradiation studies performed on anopheline mosquitoes, together with some information from other insects. No attempt is made to review all the available literature on anopheline irradiation but rather to set a baseline for future work on this subject.

## Introduction to irradiation

When biological material is irradiated, molecular bonds are broken, ions created, and free radicals formed. The free radicals attack further molecular bonds, and when DNA is damaged it can lead to the formation of dominant lethal mutations in the germ cells [16,17]. Damage to somatic cells also occurs, especially in cells undergoing mitosis. In general, damage to the germ and somatic cells increases with dose and somatic damage decreases when irradiated later in development of the insect as the number of cells undergoing division decreases. As field competitiveness is a crucial parameter, it is important to minimize the adverse effects of irradiation. Although it is generally believed that the released males need to be fully sterile, it has been suggested that more sterility can be introduced into the field population using lower radiation doses but with more competitive insects [18,19]. Moreover, reduced competitiveness can be partly overcome by increasing the ratio of sterile-to-wild insects [20].

### Radiation source and dosimetry

**Figure 1:**
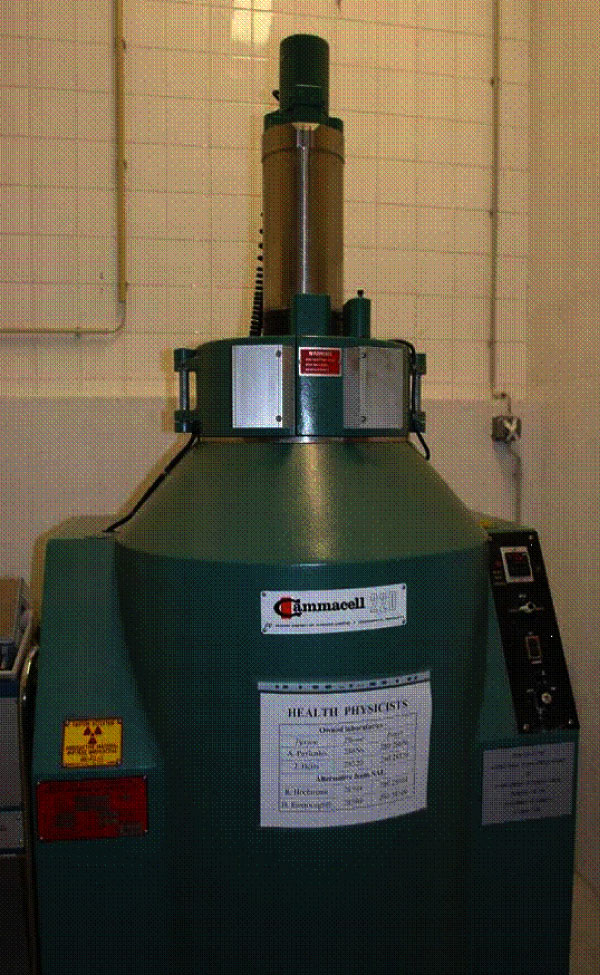
**Cobalt^60 ^irradiator**. The Gammacell 220 ^® ^(MDS Nordion, Ottawa, Canada), an example of a conventional self-shielded irradiator. In the irradiate position the sample chamber is surrounded by several rods or "pencils" of the isotope. The dose rate of the cell is determined by the activity of the source, and the absorbed dose delivered to the insects controlled by adjusting the exposure time. The sample chamber is a vertical cylinder, approximately 150 mm diameter by 200 mm tall (3.7 L) and has a typical dose uniformity ratio of about 1.7. Such self-shielded isotopic irradiators (^60^Co or less commonly ^137^Cs) are the main means of insect sterilization for SIT programmes worldwide.

For the irradiation of insects, gamma rays are usually used due to their high energy and penetration. The most common sources of gamma rays are the radioisotopes ^60^Co and ^137^Cs as both have a long half-life and emit high-energy gamma rays. ^60^Co is more easily manufactured and is therefore more often used. In conventional self-shielded irradiators (e.g. the Gammacell 220^®^, MDS Nordion, Ottawa, Canada, Figure 1), the sample chamber is surrounded by several rods or "pencils" of the isotope. The dose rate of the cell is determined by the activity of the source and the absorbed dose delivered to the insects is controlled by adjusting the exposure time [21]. The sample chamber volume of this machine is 3.7 L. The dose rate distribution in the chamber is not uniform and accordingly, insects receive different dose rates when placed at different positions in the chamber with the dose rate being most uniform towards the centre of the chamber. Besides gamma rays, X rays and accelerated electron beams can also be used to irradiate insects. X rays of appropriate energy have similar penetration as gamma rays, and they have been used in a number of studies on *Anopheles *irradiation [22-25], but the use of electron beams has not been reported.

Dosimetry is used to quantify the dose received by the irradiated insects. The selection of a suitable dosimetry system depends on several considerations including: dose range of interest, ease of handling, expertise available, cost, and uncertainty that is inherent in the system. For SIT programmes, a radiochromic film dosimetry system has been proposed [26].

### Spermatogenesis

During the successive stages of spermatogenesis, germ cells multiply and differentiate. In short, spermatogenesis includes the following developmental stages: primordial germ cells, spermatogonia (primary and secondary), spermatocytes (primary and secondary), spermatids and spermatozoa (mature sperm cells). Spermatogenesis is cystic and within one testis, cysts of different stages of development can be found. Germ cells within a single spermatocyst are more or less at the same stage of development. As spermatocysts mature, they break down and release spermatozoa into the sperm reservoir, situated in the anterior section of the testis.

Spermatogenesis occurs mainly during the larval and pupal stages but mosquito species differ in the timing of the process [27]. In *Anopheles culicifacies *[28], a small number of mature spermatozoa are already present in the sperm reservoir in the late pupal stage and many mature spermatocysts occupy the testis. During the last hour of the pupal stage and the first hours after emergence, spermatocyst maturation continues and in newly emerged males, spermatozoa make up 45% of the testis volume in *Anopheles stephensi *[29] and 41% in *An. culicifacies *[28], and spermatocysts in various stages of development (mature or not) are present [28-30]. These spermatocysts continue to mature and release sperm into the sperm reservoir during adult life, and the percentage of the testis occupied with sperm increases with age [28-30].

### Radiation sensitivity

Radiation-induced dominant lethal mutations arise as a result of chromosomal damage in the treated cells [17]. An excellent overview on the induction of dominant lethal mutations by irradiation or chemosterilization is provided by LaChance [31]. A dominant lethal mutation occurring in a germ cell does not affect the maturation of the cell into a gamete or the participation of the gamete to form the zygote but causes the death of the developing embryo [17]. In general, the earlier stages of spermatogenesis (spermatocytes and spermatogonia) are more radiosensitive than later stages (spermatids and spermatozoa) in terms of irreversible damage, and radiation can result in the death of the developing cell [21,32,33]. Irradiation of the later stages results in dominant lethal mutations in spermatozoa that lead to embryonic mortality after fertilization [32,34,35]. Irradiation also damages somatic cells, with those undergoing mitosis being the most sensitive [33]. Reduced longevity is one of the most commonly observed results of somatic damage [33]. Other effects of irradiation can be more subtle. A study in the male house fly *Musca domestica *showed that irradiation induced considerable changes in the fine structure of the fibrillar flight muscle and caused damage to the flight muscle mitochondria; the damage persisted longer in flies irradiated with higher doses [36].

### Developmental stage

To reduce somatic damage, insects should be irradiated at, or near to, the completion of their development, i.e. in mosquitoes the late pupal and adult stages. Eggs [37,38] and larvae have been irradiated [37], but this caused unacceptable mortality in the treated insects. In general, somatic damage is less pronounced in adults compared to pupae [39], although much depends on the dose and pupal age. However, handling and irradiation of pupae is considered easier due to their relative robustness compared to the adults. In *Anopheles*, under laboratory conditions, the pupal stage lasts between 25-52 h [40], depending on species and rearing conditions. Following irradiation of *Anopheles pharoensis *pupae at different ages [41], it was shown that emergence and longevity of adult mosquitoes irradiated as pupae older than 15 h did not differ from un-irradiated insects, even when the dose was high (120 Gy). The irradiation of pupae aged 1-5 h drastically decreased the emergence rate. Similar results were obtained in *Anopheles gambiae s.s*. [39]. In *Anopheles quadrimaculatus*, the irradiation of young pupae (1-4 h) with 90 Gy resulted in normal emergence [42] but longevity, measured as the percent survival after three days, was greatly reduced. The irradiation of adults following emergence at night is generally performed from the next day onwards (i.e. adults > 12 h old).

### Handling

In experimental settings, pupae can be irradiated in small wells or Petri dishes, lined with wet cotton wool covered with filter paper [41,43] allowing for the irradiation of relatively large numbers of pupae (~500). Adults, however, are much more fragile and require careful handling. Prior to irradiation, adults can be inactivated by chilling [44] which allows them to be confined in a small space within the irradiator so that dose variation can be reduced and mechanical damage to the insects minimized. In small-scale studies, adults can usually tolerate the chilling and stacking for the irradiation but in operational campaigns, very large numbers of insects will have to be irradiated and new protocols will be required. One system has been developed [39,44] that allows relatively large numbers of adults to be irradiated simultaneously (~7,000-14,000), and for recent field releases of *Aedes albopictus*, pupae were irradiated in a device that could hold ~20,000 individuals [45].

### Finding the optimal dose

To determine the optimal dose for released insects, a wide range of doses is used to generate dose response data. Initially, it is important to confine the insects in a small volume in the centre of the irradiation chamber to ensure a dose uniformity ratio of < 1.1 (where dose uniformity = highest dose/lowest dose). In operational programmes, this precision in dose distribution cannot be obtained as very large numbers of insects will need to be irradiated and the programme managers will need to define the range of acceptable doses. When determining the optimal dose, effects on sterility, longevity, and importantly, competitiveness need to be taken into account [18].

### Radiation-induced sterility

The level of sterility induced in irradiated males is measured by mating the males with un-irradiated virgin females. Eggs are then collected from females individually or *en masse *and checked for hatching. Unhatched eggs are presumed to have died due to a dominant lethal mutation (after correcting for the control sterility naturally present in the colony [46]). Control sterility in laboratory colonies was reported to be 16% in *Anopheles arabiensis *[24], 10% in *An. stephensi *and 15% in *An. gambiae s.s*. [47].

When the residual fertility is plotted on a logarithmic scale against dose, an insight into the number of dominant lethal mutations in a cell is provided [16,31]. A linear response indicates a "one-hit" relationship whereas departures from linearity indicate a "multi-hit" relationship (i.e. two or more independent events in the same cell produce a single dominant lethal event [16]).

### Longevity

Reduced longevity is often a result of radiation-induced somatic damage [33], and this must be measured, ideally, under conditions that induce stress to emphasize any differences. Specifically, male survival during the first days of adult life is important as this is the period when mating is expected to occur after release.

### Competitiveness

The ability of irradiated males to locate, compete for, and successfully couple with and inseminate the wild females is as important as their level of induced sterility [48]. Mating competition experiments are performed to study how well males are able to compete against un-irradiated males for females. Initially, competition experiments are carried out in the laboratory, but field or large outdoor cage tests must also be conducted to reveal those effects that are not evident under laboratory conditions [49]. Ideally, irradiated males are competed against wild males for wild females in a semi-field setting as wild males are likely to perform poorly under laboratory conditions.

To perform competition experiments, un-irradiated males and virgin females are introduced into a cage in a 1:1 ratio and irradiated males are introduced at equal and higher ratios. Males will compete for the females and hatching data are collected from eggs laid *en masse *or from egg batches collected from individual females that are separated after mating. When eggs are collected *en masse*, a method has been developed [50] for determining a point estimate of competitiveness for sterilized insects. This value, usually called the Fried index, can be determined provided egg hatch data are known for control (Ha) (i.e. un-irradiated females mated with un-irradiated males) and sterile (Hs) matings (i.e. un-irradiated females mated with irradiated males). The competitiveness index (C) is then estimated as C = ((Ha-Ee)/(Ee-Hs))*(N/S); where Ee is observed hatch, N = number of un-irradiated males, and S = number of irradiated males [51]. Moreover, procedures have been developed [52] to calculate an estimate of the variance of the C value where a number of replicates have been run, which permits detection of significant differences between values. The Fried index is independent of the ratio of un-irradiated to irradiated males but the variance depends strongly on the ratio and has the lowest value when half the observed matings are by irradiated males.

## Experimental work

Irradiation of *Anopheles *mosquitoes is performed with two aims. Firstly, to investigate the effect of different radiation doses on male sterility and competitiveness in the framework of an SIT programme. Such studies are often a precursor of field releases of radiation-sterilized males (see [3] and [53]). Secondly, irradiation is used to produce chromosomal rearrangements and mutations for the development of genetic sexing systems. The latter studies use a low dose so that progeny can be obtained for further analysis.

### Stage and dose range

The levels of sterility induced in several *Anopheles *species are shown in Table 1. In majority of the studies, pupae were irradiated and at a range of ages between 0-32 h. Although it is desirable to irradiate pupae as late as possible, most studies used pupae around 24 h old as this is the most convenient age for irradiation under normal laboratory rearing conditions and light regimes. Adults were irradiated from less than 24 to 96 h old. The doses administered ranged between 5-120 Gy with some studies using a wide dose-range whilst others tested fewer, depending on the goal of the study. Unfortunately, most studies do not specify whether dosimetry was used to verify the absorbed dose.

**Table 1:** Irradiation studies on male *Anopheles* pupae (P) or adults (A) and mating irradiated males (I ♂) with un-irradiated females (U ♀). Information partly from IDIDAS Database [91]

**Species**	**Stage**	**Age (hrs)**	**Dose-range**	**Induced sterility**		**Ref**.
					
				**Dose**	**I♂ × U♀**	
*An. albimanus*	P	< 24	20-80	50	84.3	[22]
					
				80	100	

*An. arabiensis*	P	23-27		120	99.6	[39]
	
	A	< 16		120	99.4	[39]
	
	A	n.r.		40	75.0	[55]
	
	P	< 24	25-100	50	76.0	[56]
					
				80	91.0	
					
				100	98.6	
	
	A	< 24	25-100	50	71.7	[56]
					
				80	96.7	
					
				100	98.1	

*An. gambiae s.s*.	P	0-7		120	99.5	[47]
					
		24-32		120	99.5	
				
	A	< 24		120	99.5	
					
		> 24		120	99.6	
	
	P	22-27		120	78-94	[39]
		
	A	< 24		80	91	
					
				120	99.3	
	
	A	24-48		45	87.4	[24]

*An. pharoensis*	P	24	50-80	50	95.8^1^	[37]
					
				80	98.7^1^	
	
	P	24		120	100^1^	[41]
	
	A	72	5-50	30	76.5	[25]
					
				50	96.8	

*An. quadrimaculatus*	P	24		118	100	[42]

*An. stephensi*	P	4-28	10-120	50	80.3*	[57]
					
				80	97.2*	
					
				120	99.1*	
	
	P	0-7		120	98.1	[47]
					
		24-32		120	98.4	
		
	A	< 24		120	98.1	
					
		> 24		120	98.2	
	
	A	< 24		120	97.5	[39]
	
	A	60	10-80	50	87.4	[58]
					
				80	96.5	

* Induced sterility was calculated from observed sterility data

^1 ^Unclear if observed or induced sterility was described

n.r. not recorded

### Sterility

The relationship between induced sterility and log dose in insects is sigmoid in form and follows the pattern of a logistic response curve. At lower doses, an approximately linear relationship between dose and induced sterility is observed while at higher doses, the curve flattens such that increasing amounts of radiation are required for proportionally smaller increases in sterility [18,19,54]. In general for *Anopheles*, doses of around 100-120 Gy induce more than 98% sterility (Table 1). At a dose of 80 Gy, more than 90% sterility is observed and at a dose of 50 Gy, sterility exceeds 70%. This is confirmed in chromosome rearrangements studies; when adult males were irradiated with 40-45 Gy, the level of sterility ranged between 75-87% [24,55]. However, a considerable amount of variation is observed when comparing sterility levels between species (Table 1; [56]). A dose of 50 Gy applied to pupae induced 76% sterility in *An. arabiensis*, 84% sterility in *Anopheles albimanus*, and 80% sterility in *An. stephensi*, while in *An. pharoensis *the level of sterility induced was 96% (Table 1). However, at the higher doses of 100-120 Gy, more than 98% sterility was induced for all species (Table 1).

In a few studies, both pupal and adult stages were irradiated, and the level of sterility induced in each was determined (Table 1). At the high dose of 120 Gy, equal levels of sterility were found for pupal and adult irradiation in *An. gambiae s.s*., although one experiment suggested pupae to be more radioresistant. In *An. arabiensis*, pupae were slightly more radioresistant at the doses of 60-80 Gy compared to adults [56]. *Anopheles stephensi *pupae and adults were irradiated in two separate studies; at 50 Gy, a higher level of induced sterility was reported for the adult irradiation. At 80 Gy, this difference was no longer observed.

### Longevity

Longevity was measured in a small number of studies and scored as daily mortality under normal rearing conditions. In most studies, differences in longevity of irradiated males compared to un-irradiated males were small with only trends being reported. Pupal irradiation in *An. pharoensis *resulted in a non-significant reduction in longevity after irradiation at doses of 100-130 Gy [41]; while in another study, a non-significant increase in longevity after irradiation with 5-70 Gy was reported [43]. In *An. arabiensis*, longevity of males irradiated as pupae with 25-100 Gy was increased, similar, or reduced compared to un-irradiated males but differences were small and longevity of males irradiated as adults was similar to un-irradiated males [56]. However, in *An. stephensi*, the longevity of males irradiated as pupae at 80 Gy was significantly reduced [57]. When pupae (22-27 h) and adults (< 24 h) of *An. gambiae s.s*. were irradiated with a high dose of 120 Gy, an increased mortality for the irradiated pupae 24 h after irradiation was reported compared to zero mortality in the irradiated adults [39].

### Mating ability

In general, the mating ability of males does not seem to be adversely affected by irradiation. The number of eggs produced by the females mated to males irradiated over a wide dose-range as pupae [22,37,41] or adults [58] is similar to un-irradiated insects; this suggests that sperm transfer from irradiated males to females was normal. Insemination rates were determined [22,56] and in *An. albimanus*, irradiated males inseminated females at an equal rate as un-irradiated males [22]. However, in the irradiated males × irradiated females group at 80 Gy, a greatly reduced insemination rate was observed [22]. In *An. arabiensis *males irradiated as pupae, a weak but significant negative correlation was observed between dose and insemination, while this was not observed after adult irradiation [56].

The ability of irradiated sperm to compete with normal sperm was assessed in *An. pharoensis *[41]. Males irradiated with 120 Gy as pupae were allowed to mate for a number of nights with virgin females after which they were removed and replaced with normal males. Females laid sterile eggs indicating that either remating did not occur or the first mating took precedence. Storage of sperm in the testes did not restore fertility when males were mated at five days after pupal irradiation with 120 Gy [41], and no recovery of fertility was observed when males irradiated as pupae or adults were remated to a second batch of virgin females after 8-9 days [39]. In addition, storage of sperm in the spermathecae from males irradiated as adults did not restore fertility when females oviposited twice [39]. A study in the honeybee, *Apis mellifera*, showed that mature sperm could not repair radiation damage even after prolonged storage of up to one year in the spermatheca [59].

### Competitiveness

Competition experiments in the laboratory have been performed in some anopheline species. Irradiated males were introduced in various ratios into rearing cages with un-irradiated males and virgin females. In most cases, the un-irradiated males and females were laboratory-reared individuals with the exception of one study [60] which used wild-caught material. Mating was allowed for a number of nights, and males were introduced soon after irradiation [42,47] or some days after emergence [57].

A selection of data on competitiveness experiments in anophelines is presented in Table 2. Most studies used only one high dose and no comparison was made between higher and lower doses with two exceptions [57,61]. Irradiation almost always had a negative impact on competitiveness of the males, especially so if the pupal stage was irradiated, resulting in higher egg hatch than would be expected if the irradiated males were equally competitive with the un-irradiated males. Egg hatch could be lowered by increasing the ratio of irradiated to un-irradiated males (Table 2) but it should be noted that where the experimental egg hatch is close to either control value (because the ratio used is too high or too low), the variance increases rapidly and the C values become meaningless [52,62].

**Table 2:** Competitiveness studies performed on *Anopheles* mosquitoes under laboratory conditions. The ratio of irradiated males (I ♂) competing with un-irradiated males (U ♂) for un-irradiated females (U ♀) is given. Insects were irradiated in air. Stage of irradiation is pupa (P) or adult (A). Where eggs were collected en masse, the competitiveness value (C) is calculated according to the Fried index ([51]; see text). In the lower part of the table data were collected by individual egg batches and C = (Sterile batches/Fertile batches) * (Number un-irradiated males/Number of sterile males)

**I♂**	**U♂**	**U♀**	**Dose (Gy)**	**Species**	**Stage**	**Age (hrs)**	**Hatch (%)**		**C value**	**Ref**.
									
							**Obs**.	**Exp**.		
1	1	1	120	*An. pharoensis*	P	20-24	39	34	0.72	[60]
						
5	1	1					20	11	0.47	
						
10	1	1					4	6	1.58	
						
15	1	1					1	4	4.40	
						
10	1	1^1^					6	7	1.03	

1	1	1	118	*An. quadrimaculatus*	P	24	74	48	0.30	[42]
						
2	1	1					85	32	0.06	
						
3	1	1					42	24	0.43	
						
4	1	1					53	19	0.20	
						
6	1	1					42	14	0.21	
						
10	1	1					28	9	0.24	

1	1	1	80	*An. stephensi*	P	4-28	54	50	0.88	[57]
					
1	1	1	120				66	50	0.51	

							**# Batches**		**C value**	
									
							**Fertile**	**Sterile**		

1	1	1	120	*An. gambiae s.s*.	P	0-7	30	7	0.24	[47]^2^
				
1	1	1			P	24-32	22	10	0.47	
				
1	1	1			A	< 24	24	26	1.06	
				
1	1	1			A	> 24	21	23	1.10	
	
1	1	1	120	*An. stephensi*	P	0-7	32	6	0.20	
				
1	1	1			P	24-32	29	9	0.32	
				
1	1	1			A	< 24	22	22	1.07	
				
1	1	1			A	> 24	19	24	1.35	

1	1	1	70	*An. arabiensis*	P	20-26	102	73	0.76	[61]^3^
			
1	1	1	120		P	20-26	141	43	0.34	
			
1	1	1	70		A	< 24	80	69	0.85	
			
1	1	1	120		A	< 24	114	59	0.54	
		
1	1	1	120		P	23-27	14	6	0.43	[39]
	
1	1	1	120	*An. gambiae s.s*.	P	22-27	20	14	0.70	
				
1	1	1			A	< 24	39	48	3.20	

^1 ^Laboratory irradiated males competed against wild males for wild females under laboratory conditions.

^2^Fertile and sterile batches averaged for the three replicates.

^3^Small cage results only.

High doses have a negative effect on competitiveness. In *An. stephensi*, males irradiated as pupae with 80 Gy were 1.7 times more competitive than males irradiated as pupae with 120 Gy [57]. In *An. arabiensis*, the use of the higher irradiation dose of 120 Gy resulted in a reduced competitiveness compared to 70 Gy for pupal and adult irradiation [61]. In *Culex quinquefasciatus*, the irradiation of adults with a dose of 50 Gy resulted in high competitiveness, but as the dose increased the competitiveness decreased [63].

Where both the pupal and the adult stages were irradiated with 120 Gy, males irradiated as adults were more competitive than males irradiated as pupae (Table 2). In *Cx. quinquefasciatus*, a dose of 80 Gy applied to pupae resulted in lower competitiveness when measured at a ratio of 1:1 compared to that for adult irradiation with a slightly lower dose of 75 Gy [7,63].

Some field studies on competitiveness of radiation-sterilized mosquitoes have been performed, although few with anophelines. In *An. quadrimaculatus*, pupae irradiated with 120 Gy and released as adults were not able to induce sterility in the target population after prolonged releases due to behaviour differences as a result of the colonization process [64]. In *Culex tarsalis*, males irradiated with 50 Gy (95% induced sterility) as adults were competitive with un-irradiated males (measured at a ratio of 1:1) from the laboratory or from field populations, in small cages indoors or large cages outdoors [65]. Males irradiated with 70 Gy were also competitive in small cages (not tested in field cages [65]). In a small release of *Cx. tarsalis *(i.e. single release of 13,000 males), males irradiated with 60 Gy were effective in inducing some sterility in the target population [66]. The continuous release of *Aedes aegypti *males (i.e. 4.6 million in 43 weeks) sterilized as pupae did not, however, result in population suppression, but the dose applied was high (110-180 Gy; [67]).

## Optimizing sterilization

Many factors influence the competitiveness of irradiated insects. Several strategies to reduce somatic damage during the irradiation process are discussed below.

### Low oxygen environment

An important factor during radiation is the oxygen level as oxygen molecules form free radicals that induce biological damage [68]. Irradiation in a low oxygen environment reduces genetic and somatic damage and consequently, higher doses are needed to induce sterility levels comparable to those induced in air, although it is often observed that competitiveness and longevity are improved despite the higher dose required. Two strategies are commonly used to reduce oxygen levels. Irradiation under hypoxia brought about by respiration of pupae kept in sealed bags is routinely performed with Mediterranean fruit fly pupae. Nitrogen has been used experimentally in tsetse [69] and routinely in Western Australia for Mediterranean fruit fly [70]. Prior to irradiation, the container that holds the insects is flushed with nitrogen for some time after which irradiation follows. Beneficial effects, i.e. long-term survival, of irradiation in a nitrogen environment were demonstrated in Mediterranean fruit fly [68] and tsetse [69,71,72].

Irradiation of mosquitoes in nitrogen has been performed in one anopheline and two culicine species. No beneficial effects of pupal radiation in *An. gambiae s.s*. [39], or pupal or adult radiation in *Cx. quinquefasciatus *[63] in nitrogen were reported. As expected, insects required much higher doses to achieve adequate sterility in nitrogen compared to air but at those higher doses, only a marginal improvement in competitiveness was observed in *Culex *adults (competitiveness was not assessed in *An. gambiae s.s*.). However in *Ae. aegypti*, irradiation in nitrogen was shown to be beneficial [73]. Competitiveness of males irradiated at 35, 70 or 100 Gy in nitrogen was equal to that of un-irradiated males, while males irradiated in air at the same doses were less competitive. Irradiation in nitrogen did increase egg hatch to some extent, but at 100 Gy, in nitrogen as well as air, 100% sterility was achieved.

### Radioprotectors

Radioprotectors are substances which when present during irradiation diminish its effects. A wide range of radioprotectors is available with various modes of action [74]. A range of protectors including amino-acids, cysteamine (aminothiol), diaminoethanetetraacetic acid (EDTA), and 2-aminoethyl isothiuronium bromide (AET) were tested on *Cx. quinquefasciatus *[75]. Pupae were soaked for a number of hours in the compounds, pre- and post-irradiation. None of the tested radioprotectors seemed to have a beneficial effect on the competitiveness or sterility of the irradiated males. However, little absorption of the radioprotector is expected in the non-ingesting pupal stage. Another protector, dimethyl sulphoxide (DMSO), ingested in the adult stage before irradiation, decreased the induction of dominant lethal mutations by X rays in *Anopheles atroparvus *[76]. However, DMSO is toxic and even at low concentrations a reduced life span was observed.

Another potential class of radioprotectors are anti-oxidants which neutralize free radicals and thus prevent damage. One of these is nordihydroguaiaretic acid (NDGA), a reducing agent that replaces the naturally present reducing agent glutathione, whose amount decreases with the age of an organism [77]. NDGA administered to the larval diet of *Ae. aegypti *increased the longevity of both sexes over un-treated controls by 42-64% [78]. The use of antioxidants has not been studied in mosquito irradiation.

### Other sterilization methods

Even though it is known that irradiation reduces competitiveness, other methods to induce sterility are controversial or not in use. Chemosterilants offer high levels of sterility with more competitive insects compared to irradiation [7,57] but their use requires special safety precautions which are difficult to implement under field conditions. Even though public opinion led to the disappearance of chemosterilants for mosquito control in the seventies, they continue to be used against other pests. In 1991, a large field trial to eradicate introduced sea lampreys *Petromyzon marinus *from the Great Lakes (USA) was initiated and lasted for several years. Radiation sterilization was considered but yielded unsatisfactory results regarding male survival and competitiveness hence, male lampreys were sterilized with the chemosterilant bisazir. However, the hazards of handling were acknowledged and alternative strategies were explored to eliminate the use of mutagenic agents [79].

Another potential class of sterilants are insect growth regulators (IGRs). IGRs have been used as sterilizing agents against housefly *M. domestica *[80], blowfly *Lucilia sericata *[81] (triflumuron) and the Mediterranean fruit fly [82] (lufenuron) using impregnated targets. The compounds were effective in inducing some sterility, although dependent on the dose administered [81]. Males could induce sterility in unexposed females, although with various degrees of success, and this was attributed to either the result of direct impairment of sperm development or transfer of active ingredient during mating to the females [81]. Also, in the tsetse fly *Glossina morsitans morsitans *sterility could be introduced in mates when males were exposed to certain juvenile hormone mimics as pupae [83]. The potential use of IGR's as a sterilant for mosquitoes remains unknown.

## Ionizing sources

Although gamma rays are the most common source of ionizing irradiation used for insect sterilization over the last decades, high-energy (5 to 10 MeV) electrons generated by accelerators and X rays can also be used [18,21]. High energy photons, both X rays and gamma rays, are gradually absorbed by the material they pass through so that the absorbed dose decays exponentially with depth into the material. The rate of the decay depends on the photon energy; at the energy of ^60^Co gamma rays it declines to half after about 23 cm in water. In contrast, electrons penetrate only a short distance before the beam is completely stopped. At an energy of 5 MeV, the penetration of an electron beam is around 4 cm in water. Even if a sample is exposed from both sides, the use of high-energy electrons, therefore, places important restrictions on the size of the irradiation canister used. Electron beams may also be converted to X rays by directing the electron beam at a high-atomic number material, such as tungsten, but the conversion efficiency, which depends on the electron beam energy is low, yielding only a few percent of the electron beam energy at 5 MeV. The recent approval of 7.5 MeV X rays from accelerators in the USA will increase the conversion efficiency available [84].

**Figure 2:**
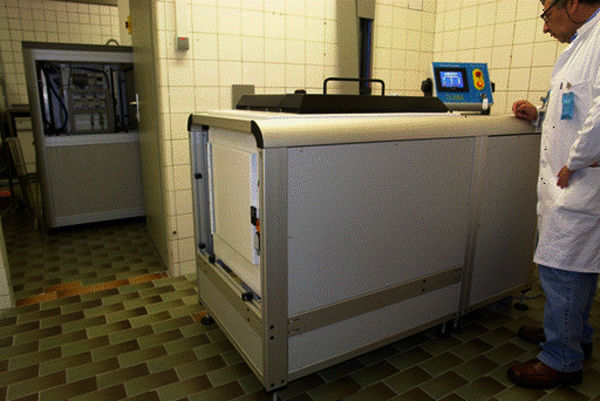
**Prototype X ray machine**. An RS2400 self-shielded cabinet 150 kV X ray irradiator (Rad Source Technologies Inc, Alpharetta, Georgia, USA), with a working volume of about 18 L divided into 5 horizontal cylinders 176 mm diameter by 150 mm long (3.6 L each) with a dose uniformity ratio of 1.3. This unit requires a 10 kW, three phase electrical supply and is cooled by chilled water or a self contained water-to-air heat exchanger (seen in the background). High power X ray units have only recently become available and are not in general use yet.

X rays can also be produced by orthovoltage tubes producing X rays with energies in the 100-500 keV range. Penetration is lower than from gamma rays or X rays produced by electron beam machines but adequate dose uniformity can be achieved by rotating the samples. X ray irradiators have suffered from low dose rates caused by the difficulty of removing the waste heat produced by the tubes, but recent advances in tube design have increased the maximum tube power substantially and dose rates of 10-15 Gy min^-1 ^are now possible in self-shielded cabinet irradiators (Figure 2) with a working volume of about 20 L (RS2400, Rad Source Technologies Inc, Alpharetta, Georgia, USA; ). These self-shielded irradiators require no special provisions for radiation security.

Isotopic irradiators have the advantage that they have a long half-life and that their dose rate is high, but the problems associated with transportation and disposal of radioactive materials are becoming increasingly difficult. A self-shielded unit, where the insect container is surrounded by several pencils, has the disadvantage that the container size is limited placing important restrictions on the throughput in SIT programmes. Furthermore, dose uniformity is poor, forcing the utilized volume to be further restricted. Panoramic irradiators are therefore more suitable as several containers can be placed around a radiation source in a large irradiation room. The containers are then rotated around their axis to achieve adequate dose uniformity [21] but dose rates tend to be lower than in self-contained irradiators.

The cost of an electron accelerator is of the order of US $1 million and power costs are high. Initial installation of the orthovoltage X ray machine is US $250,000, with a tube guaranteed for 1000 h, and a replacement tube costs about US $15,000. The installation costs of a gamma irradiator are around US $200,000 for a self-shielded cell and US $400,000 for a panoramic irradiator but the costs of transporting radio-isotopes are rising all the time as the regulations covering their transport become ever stricter.

## Discussion

In the light of all the new and exciting molecular techniques that are becoming available to create sterile insects, sterilization by irradiation might seem a little mundane. But unlike these promising techniques, irradiation has proved to be a successful, safe, and accepted way to sterilize large numbers of insects [11]. Although there are valid criticisms of SIT [85], under specific conditions, SIT could be an important tool to reduce mosquito population sizes in selected areas [86]. In addition, much criticism is directed at projects initiated decades ago whilst these days improved technologies and methods are at hand to facilitate many aspects of SIT programmes and lessons learned from the past can be applied to minimize future failure [87]. The optimal radiation dose for an SIT programme should be chosen in such a way that it balances induced sterility with competitiveness [18]. The concept of inducing 100% sterility, which was followed in the past, has led to the use of high irradiation doses, which in some insects reduced competitiveness to the extent that the target population was not sufficiently suppressed. It is now advocated that more sterility can be induced in the target population if insects are subjected to lower, partially-sterilizing doses [11,88].

The optimal developmental stage for irradiation (e.g. pupa or adult) depends on many factors including, ease of handling on a mass-production scale, competitiveness of the insect and release methodology. There was some difference in radiation sensitivity between anopheline species indicating that optimal doses in SIT programmes need to be specified for each species. The data presented here showed that irradiated anopheline males have been subjected to competitiveness assays primarily in laboratory settings and these should be complemented with studies performed under more natural conditions [49]. When determining the competitiveness of male anophelines by conventional competition assays, it has to be kept in mind that rather limited information on the courtship behaviour of wild males is available. Critical knowledge of what the important parameters are that contribute to a male's success in the field is lacking [89], although experimental work is directed increasingly to understanding male mating success [30,90]. Converting these successful traits back to measurable parameters in the laboratory is the subsequent challenge.

To reduce somatic damage caused by the irradiation process, systems such as a hypoxic environment and radiation protecting agents could be useful for mosquitoes but remain to be tested in depth. A loss of competitiveness can be overcome by increasing the number of released insects [20] but this will result in additional costs. Other methods to induce sterility, e.g. chemosterilization or transgenic approaches, are unlikely to substitute for irradiation in the near future (but see [14]). Other potential sterilants like IGRs remain to be tested in mosquitoes but would only be of use for an SIT programme if they can be applied on a mass-scale before release.

The problems with supply, usage and disposal of radioactive isotopes means that fewer new isotopic irradiators will be installed in the future, but the recent developments in X ray technology provides an adequate alternative without the security risks. The dose delivered to a large batch of insects required for mass release is not uniform so the minimum and maximum dose that the insects can receive should be determined. Quality control of the system will be crucial for a successful outcome. Dosimetry should be made part of the production process, and doses delivered to each batch need to be in the acceptable dose range. A lower dose leads to the release of insects with insufficient degree of sterility; a higher dose will produce insects with insufficient competitiveness, which will undermine the programme's efforts and overall success of the campaign. Successful quality control programmes have been implemented in ongoing SIT campaigns [26] and this knowledge can readily be transferred to other SIT programmes.

## Conclusion

At present, irradiation is the most obvious choice to sterilize mosquitoes in an SIT programme. Substantial literature on anopheline irradiation is available but should be complemented with competitiveness studies performed in a (semi-)field setting to determine the optimal dose and developmental stage for sterilization. The optimal development stage for irradiation however, also depends on the logistics of the irradiation process (e.g. the need to irradiate large numbers of insects), release methodology, and costs.

## Competing interests

The authors declare that they have no competing interests.

## Authors' contributions

The manuscript was written by MEHH, AGP and BGJK edited the manuscript.
